# Percutaneous coronary intervention in left main coronary artery disease with or without intravascular ultrasound: A meta-analysis

**DOI:** 10.1371/journal.pone.0179756

**Published:** 2017-06-22

**Authors:** Yicong Ye, Ming Yang, Shuyang Zhang, Yong Zeng

**Affiliations:** Department of Cardiology, Peking Union Medical College Hospital, Peking Union Medical College & Chinese Academy of Medical Sciences, Beijing, China; Azienda Ospedaliero Universitaria Careggi, ITALY

## Abstract

This meta-analysis compared IVUS-guided with angiography-guided PCI to determine the effect of IVUS on the mortality in patients with LM CAD. Current guidelines recommend intravascular ultrasound (IVUS)-guided percutaneous coronary intervention (PCI) in patients with left main coronary artery disease (LM CAD; Class IIa, level of evidence B). A systematic search of the MEDLINE, Embase, and Cochrane Central Register of Controlled Trials databases was conducted to identify randomized or non-randomized studies comparing IVUS-guided PCI with angiography-guided PCI in LM CAD. Ten studies (9 non-randomized and 1 randomized) with 6,480 patients were included. The primary outcome was mortality including all-cause death and cardiac death. Compared with angiography-guide PCI, IVUS-guided PCI was associated with significantly lower risks of all-cause death (risk ratio [RR] 0.60, 95% confidence interval [CI] 0.47–0.75, p<0.001), cardiac death (RR 0.47, 95% CI 0.33–0.66, p<0.001), target lesion revascularization (RR 0.43, 95% CI 0.25–0.73, p = 0.002), and in-stent thrombosis (RR 0.28, 95% CI 0.12–0.67, p = 0.004). Subgroup analyses indicated the beneficial effect of IVUS-guide PCI was consistent across different types of studies (unadjusted non-randomized studies, propensity score-matched non-randomized studies, or randomized trial), study populations (Asian versus non-Asian), and lengths of follow-up (<3 years versus ≥3 years). IVUS-guided PCI in LM CAD significantly reduced the risks of all-cause death by ~40% compared with conventional angiography-guided PCI.

**PROSPERO registration number:** CRD 42017055134.

## Introduction

A lot of evidence has indicated that percutaneous coronary intervention (PCI) is not inferior to coronary artery bypass grafting in patients with left main coronary artery disease (LM CAD), especially in those with low-moderate anatomical complexity[1–4]. Thus, current guidelines recommend PCI as an alternative to surgical revascularization in certain LM CAD groups [5].

Intravascular ultrasound has been widely used in the era of drug-eluting stents (DES) because it provides more accurate and comprehensive assessment of the structure of coronary arteries. A recent meta-analysis of 15 trials found that intravascular ultrasound (IVUS)-guided PCI significantly reduced the risk of major adverse cardiac events (MACEs) compared with angiography-guided PCI for both first and second generations of DES[6]. Another individual level meta-analysis of three randomized controlled trials (RCTs) reported that IVUS-guided PCI is associated with favorable outcomes in patients with long lesions or chronic total occlusion lesions [7].

Current guidelines recommend that IVUS be used to assess the severity and optimize the treatment of unprotect LMCA lesions (Class IIa, level of evidence B) [5]. Several new studies addressing this issue have been published in the past few years. Thus, we conducted a meta-analysis of all the studies comparing IVUS-guided and angiography-guided PCI to determine the effect of IVUS on mortality in patients with LM CAD.

## Materials and methods

The methods of this meta-analysis were pre-specified. The protocol was registered in PROSPERO (international prospective registration of systematic reviews; Registration number: CRD 42017055134). The reporting was consistent with the Preferred Reporting Items for Systematic Reviews and Meta-Analyses (PRISMA) statement (S1 File)[8].

### Data sources and searches

Systematic searches of the MEDLINE (1950 to December 2016), EMBASE (1966 to December 2016), and Cochrane Central Register of Controlled Trials (Issues 1 of 12, December 2016) were conducted to identify all studies that assessed the effect of IVUS-guided LM PCI on mortality compared with that of angiography-guided PCI. Furthermore, a manual search of the references of published reviews and meta-analyses was performed. The key words for the database searches included “left main” and “intravascular ultrasound”.

### Study selection

The eligibility of a study was determined independently by two authors (Y. Y. and Y. Z.). The inclusion criteria for this meta-analysis were: (1) study design: randomized or non-randomized; (2) study population: patients with LM CAD; (3) intervention: IVUS-guided PCI; (4) control: angiography-guided PCI; and (5) outcomes: all-cause death and/or cardiac death. Studies published as full length articles and conference abstracts were included. The exclusion criteria included: (1) non-English literature; and (2) studies included both LM and non LM CAD patients. The primary outcome of the meta-analysis was mortality (all-cause death and cardiac death), whereas the secondary outcomes included myocardial infarction [MI], target vessel revascularization [TVR], target lesion revascularization [TLR], and definite or probable in-stent thrombosis [IST].

### Data extraction

The following information was extracted independently from the included studies in a pre-specified manner by two authors (Y. Y. and Y. Z.) and included the following: (1) study design; (2) number of participants; (3) characteristics of the study population (including age, gender, mean body mass index, smoking status, hypertension, diabetes, smoking, previous cardiovascular disease, left ventricular ejection fraction, and types of LM lesions); and (4) study outcomes (all-cause death, cardiac death, MI, TVR, TLR, and IST).

### Study quality assessment

The methodological quality of the eligible studies (only full-length publications, both) was assessed independently by two authors (Y. Y. and Y. Z.) using the modified Downs and Black instrument, which can be used for both randomized and non-randomized studies [9, 10]. This instrument consists of 26 items distributed between five subscales: reporting (9 items), external validity (3 items), bias (7 items), confounding (6 items), and power (1 items). In the modified instrument, answers are scored 0 or 1, except for one item in the reporting subscale, which is scored 0 to 2. The total maximum score is 27 [10].

### Data synthesis and analysis

For randomized studies, the intention to treat data were used for analysis. For non-randomized studies, propensity score matching data were used for the meta-analysis unless they were unavailable. Pooled relative risks (RRs) were calculated using a random effects model with inverse variance method[11]. *I*^2^ statistics were used to assess the heterogeneity among the included studies[12]. Furthermore, subgroup analyses were used to explore the sources of heterogeneity with the following predefined covariates: (1) types of studies (unadjusted non-randomized studies, RCT, or propensity score-matched non-randomized studies); (2) study population (Asia or non-Asia); and (3) duration of follow-up (<3 years or ≥3 years). A cumulative meta-analysis was performed to determine the treatment effectiveness of IVUS-guided PCI by accumulation in chronological order. Sensitivity analysis was used to explore the degree to which the pooled RR for a primary outcome was affected in a conference abstract. Begg's funnel plot and Egger weighted regression statistic were used to assess the publication bias [13, 14]. All analyses were performed using RevMan software (Review Manager 5.3, The Cochrane Collaboration, Copenhagen, Denmark) and STATA software (version 11.0; Stata Corp, College Station, TX). All statistical tests were two-sided, and a p-value <0.05 was considered statistically significant.

## Results

### Study identification

**Fig 1:**
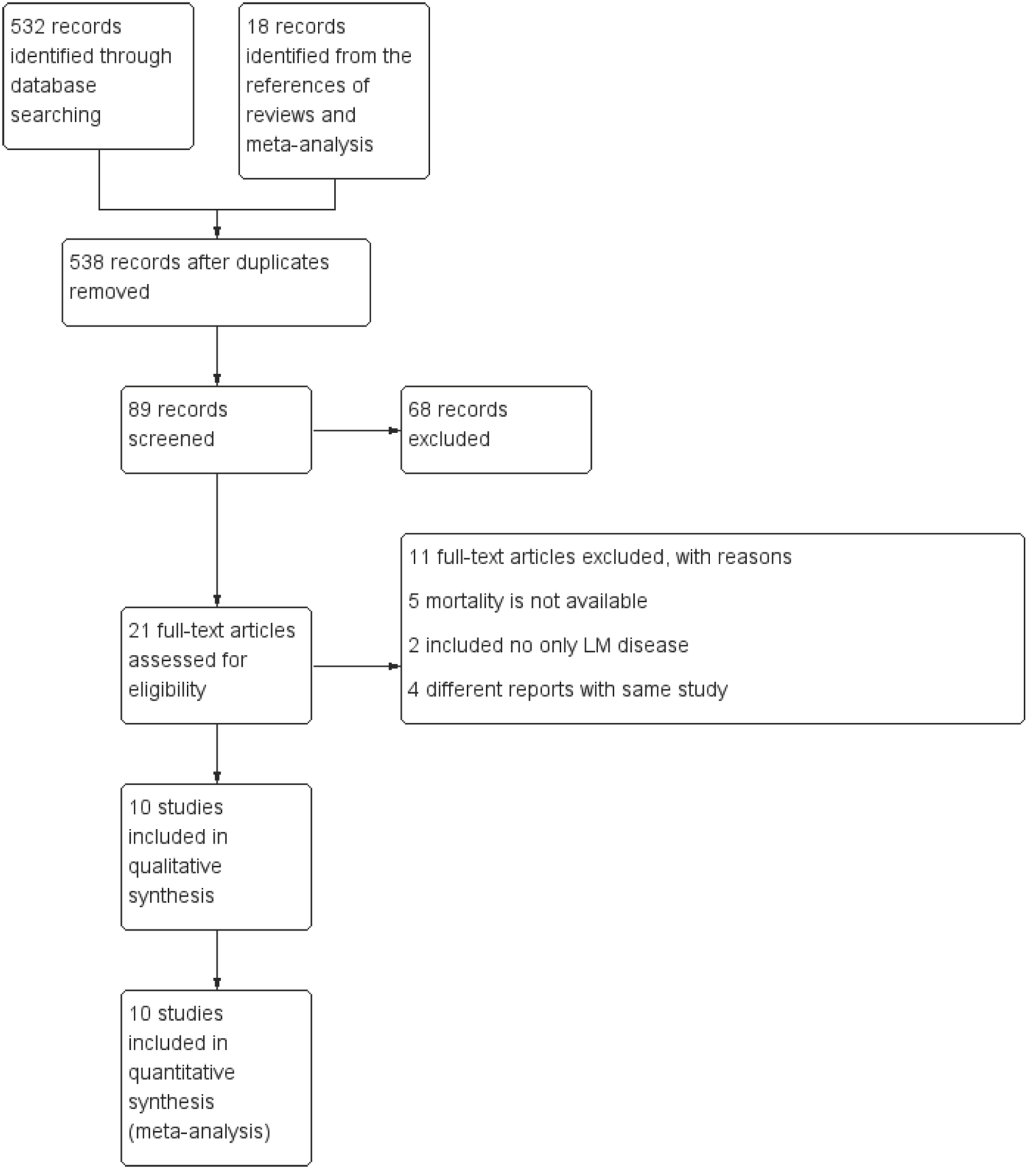
Flow chart of study selection.

The initial literature search identified 538 studies. Ten studies (1 randomized controlled trial and 9 non-randomized studies) were included in the meta-analysis based on fulfillment of the inclusion criteria [15–24]. A flowchart of study identification and screening is presented in Fig 1. Of the 10 included studies, 5 studies were reported as conference abstracts [16–19, 24]. Propensity score data were available in 4 non-randomized studies [15, 20, 21, 23]. Five studies reported both all-cause death and cardiac death [18–20, 22, 24], whereas 4 studies reported only all-cause death [15–17, 23] and 1 study reported only cardiac death[21]. The total sample size of the included patients was 6,480, of which 2,778 patients were assigned to the IVUS-guided PCI group and 3,702 patients were assigned to the angiography-guided PCI group. The information of included studies and the baseline characteristics of participants in each full-length publication are tabulated in Tables 1 and 2, respectively. The characteristics of lesion and procedure are presented in Table 3. The results of study quality assessment are summarized in S1 Table.

**Table 1:** Characteristics of the included studies.

Study	Publication type	Design	Type of data included in meta-analysis	Primary outcome in each study	Follow-up, years	All-cause mortality				Cardiac mortality			
						IVUS-guided group		Angiography-guided group		IVUS-guided group		Angiography-guided group	
						N	Death	N	Death	N	Death	N	Death
Park SJ, et al. 2009 [15]	Full-length	Non-randomized	Propensity score-matched	Death	3	145	9	145	23	/	/	/	/
Kinoshita N, et al. 2010 [16]	Abstract	Non-randomized	Unadjusted	Not specified	2	228	2	226	8	/	/	/	/
Jama A, et al.2011 [17]	Abstract	Non-randomized	Unadjusted	Death	3	111	18	184	25	/	/	/	/
Narbute I, et al. 2012 [18]	Abstract	Non-randomized	Unadjusted	Death	1	294	13	671	47	294	9	671	42
Park SH, et al. 2012 [19]	Abstract	Non-randomized	Unadjusted	Not specified	2	90	5	92	15	90	2	92	12
De La Torre Hernandez JM, et al. 2014 [20]	Full-length	Non-randomized	Propensity score-matched	Cardiac death/MI/TLR	3	505	37	505	66	505	17	505	30
Gao XF, et al. 2014 [21]	Full-length	Non-randomized	Propensity score-matched	Cardiac death/MI/TVR	1	/	/	/	/	291	5	291	15
Tan Q, et al. 2015 [22]	Full-length	Randomized	Intention to treat	Death/MI/TLR	2	61	2	62	3	61	2	62	3
Tang Y, et al. 2016 [24]	Abstract	Non-randomized	Unadjusted	Death/MI	3	713	16	1186	45	713	9	1186	31
Andell P, et al. 2017 [23]	Full-length	Non-randomized	Propensity score-matched	Death/ISR/IST	10	340	37	340	63	/	/	/	/

MI = myocardial infarction; TVR = target vessel revascularization; TLR = target lesion revasculariztion.

**Table 2:** Characteristics of participants in each included full-length publication.

Study	Age, years	Male, %	HTN, %	DM, %	Smoker, %	Prior PCI, %	Prior MI, %	CRF, %	ACS, %	LVEF, %	LM distal, %
Park SJ, et al. 2009 [15]	64.8	70.9	54.7	33.1	22.4	22.1	8.5	3.0	61.2	61.4	53.0
De La Torre Hernandez JM, et al. 2014 [20]	66.5	79.4	66	35.4	30.6	21.6	24.9	6.5	60.0	55.1	44.2
Gao XF, et al. 2014 [21]	66.7	78.7	72.1	33.6	33.6	17.6	18.0%	29.7	/	57.4	86.4
Tan Q, et al. 2015 [22]	76.2	65.9	68.3	31.7	45.5	18.9	/	/	68.3	54.3	53.7
Andell P, et al. 2017 [23]	71.5	72.4	73.5	24.6	13.6	33.3	34.7	3.2	64.7	/	/

HTN = hypertension; DM = diabetes mellitus; PCI = percutaneous coronary intervention; MI = myocardial infarction; CRF = chronic renal failure; ACS = acute coronary syndrome; LVEF = left ventricular ejection fraction; LM = left main.

**Table 3:** Characteristics of lesions and procedure in each included full-length publication.

Study	Intervention	LM lesion		Extent of diseased vessel				Complex stenting
		Ostium/shaft	Distal	LM only	LM+ single VD	LM+ 2VD	LM+ 3VD	
Park SJ, et al. 2009 [15]	IVUS-guided	46.3	53.7	13.9	26.4	29.4	30.4	22.4
	Angiography-guided	47.8	52.2	14.4	22.4	30.9	32.3	22.4
De La Torre Hernandez JM, et al. 2014 [20]	IVUS-guided	56.3	43.7	/	/	31.7	31.9	12.5
	Angiography-guided	55.3	44.7	/	/	33.2	29.5	12.2
Gao XF, et al. 2014 [21]	IVUS-guided	14.2	85.8	/	/	/	/	45.7
	Angiography-guided	13.1	86.9	/	/	/	/	41.2
Tan Q, et al. 2015 [22]	IVUS-guided	47.5	52.5	11.5	23.0	39.3	26.2	40.3
	Angiography-guided	45.2	54.8	16.1	21.0	35.5	27.4	41.9
Andell P, et al. 2017 [23]	IVUS-guided	30.9	69.1	9.4	35.0	34.7	20.9	/
	Angiography-guided	30.9	69.1	10.0	36.2	34.7	19.1	/

*p* for all intergroup difference in each study > 0.05; LM = left main; VD = vessel disease.

### Quantitative data analysis

**Fig 2:**
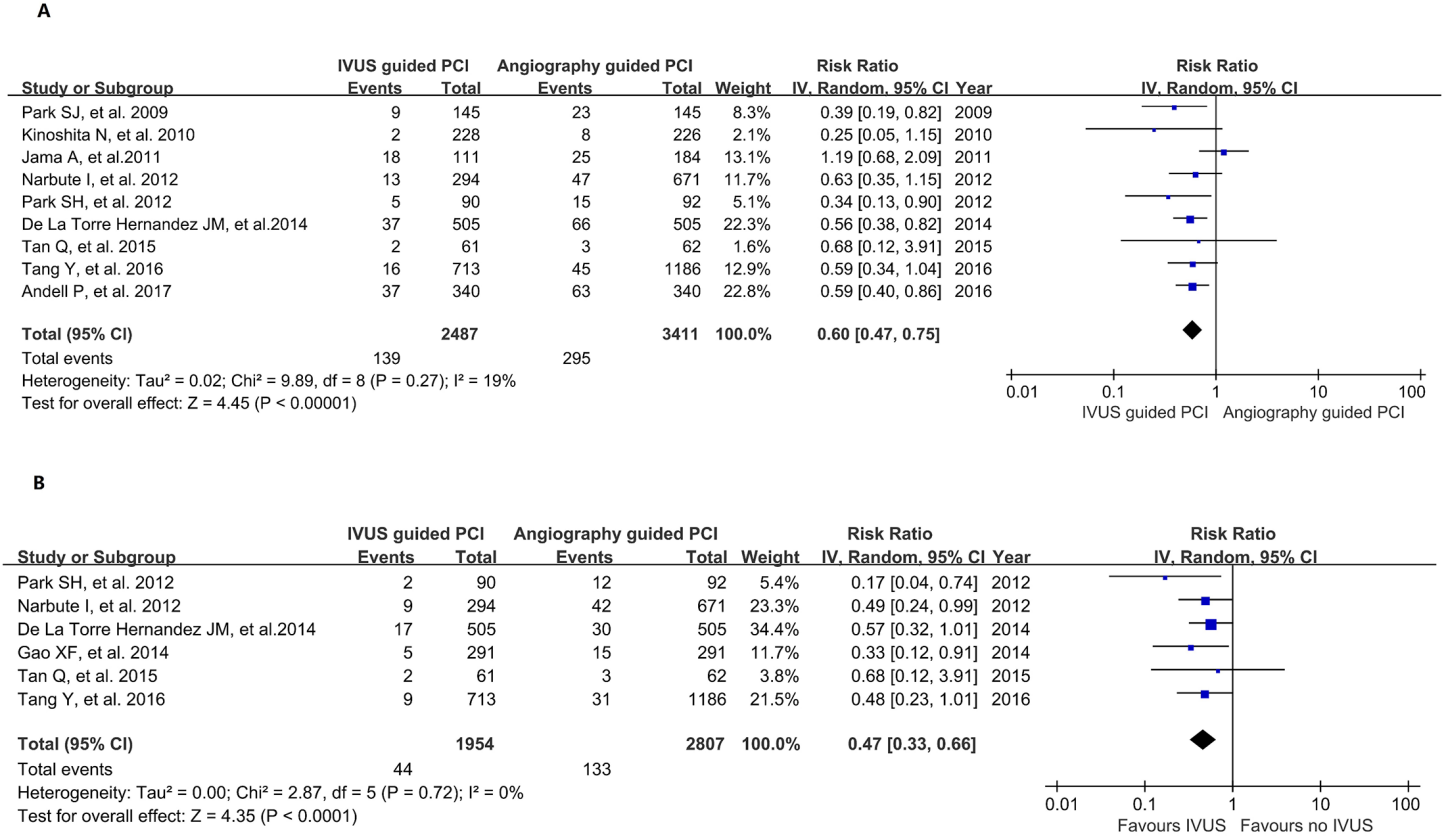
Forest plot of primary outcomes; (A) all-cause death; (B) cardiac death.

For the primary outcomes, IVUS-guided PCI significantly reduced the risk of all-cause death compared with angiography-guided PCI (RR 0.60, 95% confidence interval [CI] 0.47–0.75, p<0.001; Fig 2A) with moderate heterogeneity (χ^2^ = 9.89, I^2^ = 19%, p for heterogeneity = 0.27). In addition, IVUS-guided PCI was associated with a lower risk of cardiac death compared with angiography-guided PCI (RR 0.47, 95% CI 0.33–0.66, p<0.001; Fig 2B). There was no statistically significant heterogeneity across the included studies (χ^2^ = 2.87, I^2^ = 0%, p for heterogeneity = 0.72).

For the secondary outcomes, IVUS-guided PCI was associated with lower risks of TLR (RR 0.43, 95% CI 0.25–0.73, p = 0.002; I^2^ = 0%, p for heterogeneity = 0.53) and definite or probable IST (RR 0.28, 95% CI 0.12–0.67, p = 0.004; I^2^ = 5%, *p* for heterogeneity = 0.37) compared with angiography-guided PCI. However, there were no differences in the risks of MI and TVR between the two groups (Table 4).

**Table 4:** Pooled results for secondary outcomes.

Secondary outcome	Number of studies	IVUS-guided group	Angiography-guided group	RR	95% CI	p for RR	I^2^	p for heterogeneity
Myocardial infarction	7	114/1916	181/2465	0.8	0.61–1.06	0.12	22%	0.26
Target vessel revascularization	6	147/1972	191/2445	0.89	0.66–1.20	0.44	47%	0.09
Target lesion revascularization	3	18/442	43/445	0.43	0.25–0.73	0.002	0%	0.53
In-stent thrombosis	4	7/1197	37/1198	0.28	0.12–0.67	0.004	5%	0.37

IVUS = intravascular ultrasound; RR = risk ratio; CI = confidence interval

**Fig 3:**
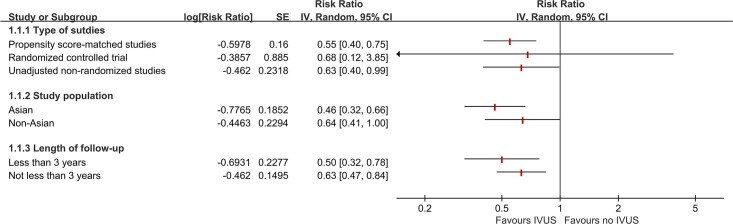
Results of subgroup analyses.

**Fig 4:**
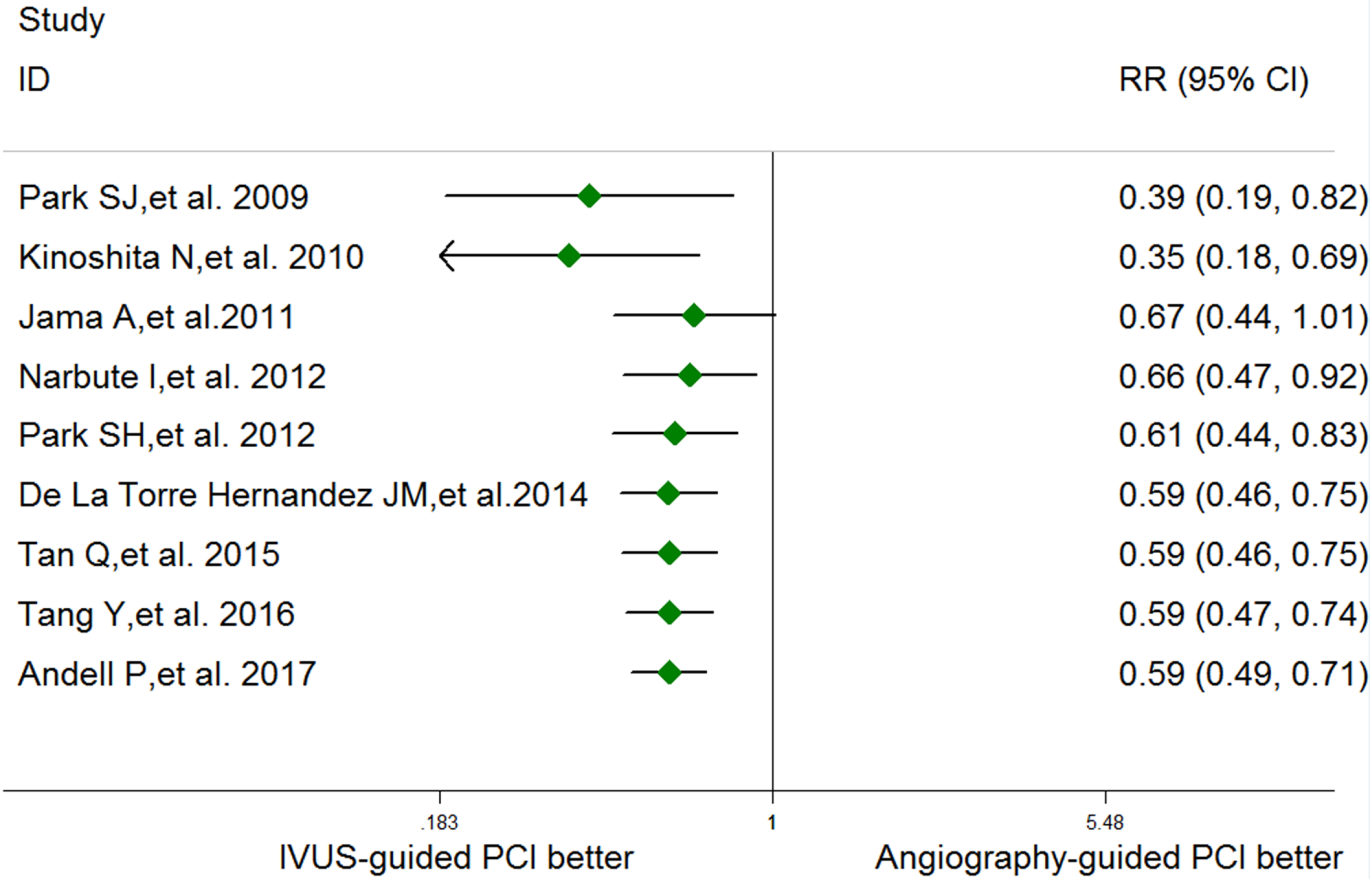
Results of cumulative meta-analysis in chronological order.

In the pre-specified subgroup analyses, there were no significant interactions between subgroups regarding the types of study, study population, or length of follow-up (all *p* for subgroup differences >0.05; Fig 3). Cumulative meta-analysis indicated that there was a consistent beneficial effect of IVUS-guided PCI since 2012 (Fig 4). Sensitivity analysis showed that the pooled RRs, excluding conference abstract data, were comparable (RR 0.56, 95% CI 0.44–0.70, p<0.001; I^2^ = 0%, p for heterogeneity = 0.90), indicating the final results were not affected by inclusion of conference abstracts.

Both Begg’s test (p = 0.532) and Egger weighted regression statistic (p = 0.587) suggested no significant publication bias across the studies.

## Discussion

Our meta-analysis of 10 studies indicated that PCI under IVUS guidance for LM CAD could reduce the risk of all-cause mortality by 40% and cardiac death by 53% compared with conventional angiography-guided PCI. In addition, IVUS-guided PCI in LM CAD was associated with lower risks of TLR and IST.

Park et al. first found that guidance with IVUS may optimize the immediate outcome achieved with a larger lumen diameter in comparison to not using IVUS guidance in selected patients receiving unprotected LM stenting [25]. Agostoni et al. first assessed the effect of IVUS on the early clinical outcomes in 58 patients who underwent PCI for LM CAD. Due to the small sample size, there study was unable to report a statistical difference in the risk of MACEs (8% in IVUS group versus 20% in non IVUS group, p = 0.18) [26]. Park et al’s study from the MANI-COMPARE registry was the first to demonstrate the possible benefits of IVUS guidance for reducing long-term mortality associated with PCI for unprotected LM CAD[15], and this study is the major reference for the recommendation in current guidelines. Indeed, several studies have been published since then, and most of them reported a similar conclusion. Our cumulative meta-analysis indicated that the beneficial effect of IVUS-guidance in LMCA PCI has become consistent since the publication of Narbute *et al*. in 2012. This may justify modification of the level of evidence in guidelines.

To date, there has been only one small randomized controlled trial comparing IVUS-guided with angiography-guided PCI in LM CAD. Most sources of evidence came from non-randomized studies. Because the characteristics of participants in the two groups would be quite different in this situation, controlling for confounding factors becomes one of the major issues when conducting a meta-analysis of non-randomized studies[27]. Propensity score matching is a statistical technique as powerful as regression for confounder adjustment when estimating a treatment effect[28]. In the subgroup analyses, pooled results from propensity score-matched studies and randomized controlled trials were found to be similar with those from unadjusted non-randomized studies, indicating that the conclusion was less likely to be compromised by the confounding effect of the non-randomized studies.

The role of IVUS in complex PCI have been investigated by several studies. Previous studies have reported that IVUS is a valuable tool for recanalization of the chronic total occlusion (CTO) [29, 30] and IVUS-guided CTO intervention have been proved to be associated with lower 12-month major adverse cardiac event rate [31]. A recent meta-analysis of eight RCT have confirmed the IVUS-guided PCI could significantly reduce the risk of major adverse cardiac events and target lesion/vessel revascularization in patients with complex lesion, such as CTO and long coronary lesions[32]. The mechanisms by which IVUS-guided PCI improves survival in LM CAD are still uncertain and may be associated with pre-PCI and post-PCI assessments.

Although angiography was considered the ‘gold standard’ for coronary artery assessment, the severity of atherosclerosis might be misjudged due to significant inter- and intra-observer variability [33–35]. In a study of IVUS by Oviedo et al., the positive predictive values of angiography (diameter stenosis >50% or “1” in the Medina classification) for identifying an IVUS plaque burden >70% or a reduction in minimum lumen area (<4.0 mm^2^ for the ostial LAD or LCX artery and <6.0 mm^2^ for the distal LMCA) were only 35.1% and 56.7%, respectively [36]. Thus, angiographic assessment of LMCA bifurcation lesions was rarely accurate, which could lead to use of undesirable strategies. Although there is no evidence from RCT, the single-stent technique could be considered as the default strategy for bifurcation LM lesions in selected patients, such as insignificant ostial LCX stenosis or non-left dominant coronary system [37, 38]. IVUS provides more accurate information of the disease status of the distal LM complex, especially the LCX ostium. Kang et al found that an IVUS-derived minimal lumen of >3.7 mm^2^ or plaque burden of <56% in the LCX ostium can exclude functional LCX compromise (fractional flow reserve <0.80) after main vessel stenting with single stent technique [39].

For post-PCI assessment, a comprehensive study of IVUS by Kang et al. found that the stent area in the LMCA was associated with in-stent restenosis (ISR) and clinical outcomes [40]. The cutoffs that best predicted ISR were 5.0 mm^2^, 6.3 mm^2^, 7.2 mm^2^, and 8.2 mm^2^ for the ostial LCX, ostial LAD, polygon of confluence, and distal LM, respectively. Clinical outcomes might be improved by IVUS optimization during LMCA stenting procedures with these criteria [40]. Furthermore, the results of this meta-analysis have suggested a reduction in the risk of IST with IVUS-guided PCI. It is reported that when performing double-kissing (DK) crush, IVUS-guided procedure could improve the procedural quality (less malaposition, edge dissection, and stent expansion) and was associated with a decrease IST, resulting in a significant decrement of ST-elevation MI [41].

There were several limitations in the current study. First, this was a study-level meta-analysis instead of a patient-level meta-analysis, and thus, we could not assess the effect of all different factors on the conclusion. Second, this meta-analysis included mainly non-randomized studies. We believe these conclusion could provide fundamental information for the future RCT. Third, Although our subgroup analysis of propensity score-matched studies confirmed the conclusion, unmeasured confounders may have influenced the outcomes. Finally, some important characteristics of the patients such as SANTAX scores were not reported in the included studies. Also, the included conference abstracts provided limited information on the patient and lesion characteristics.

## Conclusions

This meta-analysis suggested that IVUS-guided PCI is superior to angiography-guided PCI in LMCA PCI, based on reductions in the risks of both all-cause and cardiac death. Still, a larger scale RCT should be conducted to confirm these conclusions.

## Supporting information

S1 Table Quality assessment of the included full-length publications using the modified Down and Black instrument. (DOC)

S1 File PRISMA checklist. (PDF)

## References

[1] Capodanno D, Stone GW, Morice MC, Bass TA, Tamburino C. Percutaneous coronary intervention versus coronary artery bypass graft surgery in left main coronary artery disease: a meta-analysis of randomized clinical data. Journal of the American College of Cardiology. 2011;58(14):1426–32. doi: 10.1016/j.jacc.2011.07.005 . 21939824 10.1016/j.jacc.2011.07.005

[2] Stone GW, Sabik JF, Serruys PW, Simonton CA, Genereux P, Puskas J, et al Everolimus-Eluting Stents or Bypass Surgery for Left Main Coronary Artery Disease. The New England journal of medicine. 2016;375(23):2223–35. doi: 10.1056/NEJMoa1610227 . 27797291 10.1056/NEJMoa1610227

[3] Ahn JM, Roh JH, Kim YH, Park DW, Yun SC, Lee PH, et al Randomized Trial of Stents Versus Bypass Surgery for Left Main Coronary Artery Disease: 5-Year Outcomes of the PRECOMBAT Study. Journal of the American College of Cardiology. 2015;65(20):2198–206. doi: 10.1016/j.jacc.2015.03.033 . 25787197 10.1016/j.jacc.2015.03.033

[4] Morice MC, Serruys PW, Kappetein AP, Feldman TE, Stahle E, Colombo A, et al Five-year outcomes in patients with left main disease treated with either percutaneous coronary intervention or coronary artery bypass grafting in the synergy between percutaneous coronary intervention with taxus and cardiac surgery trial. Circulation. 2014;129(23):2388–94. doi: 10.1161/CIRCULATIONAHA.113.006689 . 24700706 10.1161/CIRCULATIONAHA.113.006689

[5] Authors/Task Force m, Windecker S, Kolh P, Alfonso F, Collet JP, Cremer J, et al 2014 ESC/EACTS Guidelines on myocardial revascularization: The Task Force on Myocardial Revascularization of the European Society of Cardiology (ESC) and the European Association for Cardio-Thoracic Surgery (EACTS)Developed with the special contribution of the European Association of Percutaneous Cardiovascular Interventions (EAPCI). European heart journal. 2014;35(37):2541–619. doi: 10.1093/eurheartj/ehu278 . 25173339 10.1093/eurheartj/ehu278

[6] Nerlekar N, Cheshire CJ, Verma KP, Ihdayhid AR, McCormick LM, Cameron JD, et al Intravascular ultrasound guidance improves clinical outcomes during implantation of both first and second-generation drug-eluting stents: a meta-analysis. EuroIntervention: journal of EuroPCR in collaboration with the Working Group on Interventional Cardiology of the European Society of Cardiology. 2016 doi: 10.4244/EIJ-D-16-00769 . 27840327 10.4244/EIJ-D-16-00769

[7] Shin DH, Hong SJ, Mintz GS, Kim JS, Kim BK, Ko YG, et al Effects of Intravascular Ultrasound-Guided Versus Angiography-Guided New-Generation Drug-Eluting Stent Implantation: Meta-Analysis With Individual Patient-Level Data From 2,345 Randomized Patients. JACC Cardiovascular interventions. 2016;9(21):2232–9. doi: 10.1016/j.jcin.2016.07.021 . 27744039 10.1016/j.jcin.2016.07.021

[8] Liberati A, Altman DG, Tetzlaff J, Mulrow C, Gotzsche PC, Ioannidis JP, et al The PRISMA statement for reporting systematic reviews and meta-analyses of studies that evaluate health care interventions: explanation and elaboration. Journal of clinical epidemiology. 2009;62(10):e1–34. doi: 10.1016/j.jclinepi.2009.06.006 . 19631507 10.1016/j.jclinepi.2009.06.006

[9] Downs SH, Black N. The feasibility of creating a checklist for the assessment of the methodological quality both of randomised and non-randomised studies of health care interventions. Journal of epidemiology and community health. 1998;52(6):377–84. ; 9764259 10.1136/jech.52.6.377 PMC1756728

[10] Trac MH, McArthur E, Jandoc R, Dixon SN, Nash DM, Hackam DG, et al Macrolide antibiotics and the risk of ventricular arrhythmia in older adults. CMAJ: Canadian Medical Association journal = journal de l'Association medicale canadienne. 2016;188(7):E120–9. doi: 10.1503/cmaj.150901 ; 26903359 10.1503/cmaj.150901 PMC4835295

[11] DerSimonian R, Laird N. Meta-analysis in clinical trials. Controlled clinical trials. 1986;7(3):177–88. . 3802833 10.1016/0197-2456(86)90046-2

[12] Higgins JP, Thompson SG, Deeks JJ, Altman DG. Measuring inconsistency in meta-analyses. BMJ (Clinical research ed). 2003;327(7414):557–60. Epub 2003/09/06. doi: 10.1136/bmj.327.7414.557 ; 12958120 10.1136/bmj.327.7414.557 PMC192859

[13] Begg CB, Mazumdar M. Operating characteristics of a rank correlation test for publication bias. Biometrics. 1994;50(4):1088–101. . 7786990

[14] Egger M, Davey Smith G, Schneider M, Minder C. Bias in meta-analysis detected by a simple, graphical test. BMJ (Clinical research ed). 1997;315(7109):629–34. Epub 1997/10/06. ; 9310563 10.1136/bmj.315.7109.629 PMC2127453

[15] Park SJ, Kim YH, Park DW, Lee SW, Kim WJ, Suh J, et al Impact of intravascular ultrasound guidance on long-term mortality in stenting for unprotected left main coronary artery stenosis. Circulation: Cardiovascular Interventions. 2009;2(3):167–77. 20031713 10.1161/CIRCINTERVENTIONS.108.799494

[16] Kinoshita N, Ohota K, Yamada T, Miyai N, Nakamura R, Irie H, et al Clinical long-term outcomes after DES stenting with or without intravascular ultrasound guidance. American Journal of Cardiology. 2010;105(9):59B.20102891

[17] Jama A, Conrotto F, Lennon R, Lerman A. The clinical impact of intravascular ultrasound in patients undergoing implantation of drug-eluting stents in the left main. J Am Coll Cardiol. 2011;58(20):B167.

[18] Narbute I, Kumsars I, Trusinskis K, Sondore D, Jegere S, Latkovskis G, et al Better one-year survival in consecutive unprotected left main patients with cutting balloon pre-dilatation and IVUS guidance. EuroIntervention: journal of EuroPCR in collaboration with the Working Group on Interventional Cardiology of the European Society of Cardiology. 2012;8:N101.

[19] Park SH, Rha SW, Cho AR, Lee HG, Lee SW, Shin WY, et al Impact of intravascular ultrasound guided left main intervention with drug-eluting stents on 2-year clinical outcomes. American Journal of Cardiology. 2012;109(7):123S.

[20] De La Torre Hernandez JM, Alonso JAB, Hospital JAG, Manterola FA, Camarero TG, De Carlos FG, et al Clinical impact of intravascular ultrasound guidance in drug-eluting stent implantation for unprotected left main coronary disease: Pooled analysis at the patient-level of 4 registries. JACC: Cardiovascular Interventions. 2014;7(3):244–54. doi: 10.1016/j.jcin.2013.09.014 24650399 10.1016/j.jcin.2013.09.014

[21] Gao XF, Kan J, Zhang YJ, Zhang JJ, Tian NL, Ye F, et al Comparison of one-year clinical outcomes between intravascular ultrasound-guided versus angiography-guided implantation of drug-eluting stents for left main lesions: A single-center analysis of a 1,016-patient cohort. Patient Preference and Adherence. 2014;8:1299–309. doi: 10.2147/PPA.S65768 25278749 10.2147/PPA.S65768 PMC4179827

[22] Tan Q, Wang Q, Liu D, Zhang S, Zhang Y, Li Y. Intravascular ultrasound-guided unprotected left main coronary artery stenting in the elderly. Saudi Medical Journal. 2015;36(5):549–53. doi: 10.15537/smj.2015.5.11251 25935174 10.15537/smj.2015.5.11251 PMC4436750

[23] Andell P, Karlsson S, Mohammad MA, Gotberg M, James S, Jensen J, et al Intravascular Ultrasound Guidance Is Associated With Better Outcome in Patients Undergoing Unprotected Left Main Coronary Artery Stenting Compared With Angiography Guidance Alone. Circulation Cardiovascular interventions. 2017;10(5). doi: 10.1161/CIRCINTERVENTIONS.116.004813 . 28487356 10.1161/CIRCINTERVENTIONS.116.004813

[24] Tang Y, Tian J, Guan C, Wang W, Zhang K, Chen J, et al TCT-555 Intravascular Ultrasound Guidance Improves the Long-term Prognosis in Patients with Unprotected Left Main Coronary Artery Disease Undergoing Percutaneous Coronary Intervention. Journal of the American College of Cardiology. 2016;68(18S):B224 doi: 10.1016/j.jacc.2016.09.693 10.1038/s41598-017-02649-5 PMC5443793 28539596

[25] Park SJ, Hong MK, Lee CW, Kim JJ, Song JK, Kang DH, et al Elective stenting of unprotected left main coronary artery stenosis: Effect of debulking before stenting and intravascular ultrasound guidance. J Am Coll Cardiol. 2001;38(4):1054–60. 11583882 10.1016/s0735-1097(01)01491-7

[26] Agostoni P, Valgimigli M, Van Mieghem CAG, Rodriguez-Granillo GA, Aoki J, Ong ATL, et al Comparison of early outcome of percutaneous coronary intervention for unprotected left main coronary artery disease in the drug-eluting stent era with versus without intravascular ultrasonic guidance. American Journal of Cardiology. 2005;95(5):644–7. doi: 10.1016/j.amjcard.2004.10.042 15721110 10.1016/j.amjcard.2004.10.042

[27] Valentine JC, Thompson SG. Issues relating to confounding and meta-analysis when including non-randomized studies in systematic reviews on the effects of interventions. Research synthesis methods. 2013;4(1):26–35. doi: 10.1002/jrsm.1064 . 26053537 10.1002/jrsm.1064

[28] Shah BR, Laupacis A, Hux JE, Austin PC. Propensity score methods gave similar results to traditional regression modeling in observational studies: a systematic review. Journal of clinical epidemiology. 2005;58(6):550–9. doi: 10.1016/j.jclinepi.2004.10.016 . 15878468 10.1016/j.jclinepi.2004.10.016

[29] Furuichi S, Airoldi F, Colombo A. Intravascular ultrasound-guided wiring for chronic total occlusion. Catheterization and cardiovascular interventions: official journal of the Society for Cardiac Angiography & Interventions. 2007;70(6):856–9. 17932882 10.1002/ccd.21219

[30] Park Y, Park HS, Jang GL, Lee DY, Lee H, Lee JH, et al Intravascular ultrasound guided recanalization of stumpless chronic total occlusion. International journal of cardiology. 2011;148(2):174–8. doi: 10.1016/j.ijcard.2009.10.052 . 19942305 10.1016/j.ijcard.2009.10.052

[31] Kim BK, Shin DH, Hong MK, Park HS, Rha SW, Mintz GS, et al Clinical Impact of Intravascular Ultrasound-Guided Chronic Total Occlusion Intervention With Zotarolimus-Eluting Versus Biolimus-Eluting Stent Implantation: Randomized Study. Circulation Cardiovascular interventions. 2015;8(7):e002592 doi: 10.1161/CIRCINTERVENTIONS.115.002592 . 26156151 10.1161/CIRCINTERVENTIONS.115.002592

[32] Bavishi C, Sardar P, Chatterjee S, Khan AR, Shah A, Ather S, et al Intravascular ultrasound-guided vs angiography-guided drug-eluting stent implantation in complex coronary lesions: Meta-analysis of randomized trials. American heart journal. 2017;185:26–34. doi: 10.1016/j.ahj.2016.10.008 . 28267472 10.1016/j.ahj.2016.10.008

[33] Fisher LD, Judkins MP, Lesperance J, Cameron A, Swaye P, Ryan T, et al Reproducibility of coronary arteriographic reading in the coronary artery surgery study (CASS). Catheterization and cardiovascular diagnosis. 1982;8(6):565–75. . 7151153 10.1002/ccd.1810080605

[34] Zir LM, Miller SW, Dinsmore RE, Gilbert JP, Harthorne JW. Interobserver variability in coronary angiography. Circulation. 1976;53(4):627–32. . 1253383 10.1161/01.cir.53.4.627

[35] Detre KM, Wright E, Murphy ML, Takaro T. Observer agreement in evaluating coronary angiograms. Circulation. 1975;52(6):979–86. . 1102142 10.1161/01.cir.52.6.979

[36] Oviedo C, Maehara A, Mintz GS, Araki H, Choi SY, Tsujita K, et al Intravascular ultrasound classification of plaque distribution in left main coronary artery bifurcations: where is the plaque really located? Circulation Cardiovascular interventions. 2010;3(2):105–12. doi: 10.1161/CIRCINTERVENTIONS.109.906016 . 20197513 10.1161/CIRCINTERVENTIONS.109.906016

[37] Roh JH, Santoso T, Kim YH. Which technique for double stenting in unprotected left main bifurcation coronary lesions? EuroIntervention: journal of EuroPCR in collaboration with the Working Group on Interventional Cardiology of the European Society of Cardiology. 2015;11 Suppl V:V125–8. doi: 10.4244/EIJV11SVA28 . 25983145 10.4244/EIJV11SVA28

[38] Park SJ, Ahn JM, Foin N, Louvard Y. When and how to perform the provisional approach for distal LM stenting. EuroIntervention: journal of EuroPCR in collaboration with the Working Group on Interventional Cardiology of the European Society of Cardiology. 2015;11 Suppl V:V120–4. doi: 10.4244/EIJV11SVA27 . 25983144 10.4244/EIJV11SVA27

[39] Kang SJ, Ahn JM, Kim WJ, Lee JY, Park DW, Lee SW, et al Functional and morphological assessment of side branch after left main coronary artery bifurcation stenting with cross-over technique. Catheterization and cardiovascular interventions: official journal of the Society for Cardiac Angiography & Interventions. 2014;83(4):545–52. 23765939 10.1002/ccd.25057

[40] Kang SJ, Ahn JM, Song H, Kim WJ, Lee JY, Park DW, et al Comprehensive intravascular ultrasound assessment of stent area and its impact on restenosis and adverse cardiac events in 403 patients with unprotected left main disease. Circulation Cardiovascular interventions. 2011;4(6):562–9. doi: 10.1161/CIRCINTERVENTIONS.111.964643 . 22045969 10.1161/CIRCINTERVENTIONS.111.964643

[41] Chen SL, Ye F, Zhang JJ, Tian NL, Liu ZZ, Santoso T, et al Intravascular ultrasound-guided systematic two-stent techniques for coronary bifurcation lesions and reduced late stent thrombosis. Catheterization and cardiovascular interventions: official journal of the Society for Cardiac Angiography & Interventions. 2013;81(3):456–63. 22899562 10.1002/ccd.24601

