# Supplementary material for: Percutaneous coronary intervention in left main coronary artery disease with or without intravascular ultrasound: A meta-analysis
Source: PLoS One. 2017 Jun 22;12(6):e0179756. doi: 10.1371/journal.pone.0179756 (PMC5481000; doi:10.1371/journal.pone.0179756)
Supplement: S1 Table — (DOC) [file pone.0179756.s001.doc]

S1 Table. Quality assessment of the included full-length publications using the modified Down and Black instrument

| Study | Reporting | External validity | Internal validity-Bias | Internal validity-Confounding | Power |
| --- | --- | --- | --- | --- | --- |
| Park SJ, et al. 2009 | 9 | 2 | 4 | 3 | 0 |
| De La Torre Hernandez JM, et al.2014 | 9 | 2 | 4 | 3 | 0 |
| Gao XF, et al. 2014 | 10 | 2 | 5 | 3 | 0 |
| Tan Q, et al. 2015 | 10 | 2 | 4 | 5 | 0 |
| Andell, et al. 2017 | 10 | 2 | 4 | 5 | 0 |
